# Spots and dots in the bones

**DOI:** 10.1186/1470-7330-15-S1-O4

**Published:** 2015-10-02

**Authors:** Philippa Tyler

**Affiliations:** 1 The Royal National Orthopaedic Hospital, Stanmore, Middlesex, HA7 4LP, UK; 2 Institute of Orthopaedics, University College London, UK

## 

Cancer imaging is frequently at the cutting edge of new imaging techniques which are often rapidly incorporated into routine use.

Skeletal metastatic disease is a frequent complication of neoplastic conditions, and results in specific challenges to the general radiologist and specialist oncological radiologist alike.

Non-neoplastic conditions and normal variants may simulate skeletal metastases, and the radiologist must recognise such cases and avoid over-investigation and unnecessary treatment.

This lecture will briefly review standard imaging techniques and demonstrate normal appearances, normal variants and non-neoplastic lesions that mimic primary and secondary skeletal malignancy, and will then review a spectrum of malignancy-associated bone lesions with the use of standard and more specialised imaging techniques, including PET MRI, PET CT and diffusion weighted imaging,.

Expected post treatment imaging findings, and treatment-associated complications will also be discussed.
